# Neurological and mental health in the era of climate change: mechanisms, clinical impacts, and adaptation

**DOI:** 10.3389/fpubh.2025.1630975

**Published:** 2025-09-11

**Authors:** Pablo S. Martínez Lozada, Jose E. Leon-Rojas

**Affiliations:** ^1^NeurALL Research Group, Quito, Ecuador; ^2^Cerebro, Emoción y Conducta (CEC) Research Group, Escuela de Medicina, Universidad de las Américas (UDLA), Quito, Ecuador

**Keywords:** climate change, public health, neurological and neurodevelopmental diseases, psychiatric disease, mental health, environmental health

## Abstract

Climate change has become a global health emergency in recent decades, with far-reaching effects on neurological and psychiatric health; however, their relationship remains poorly understood. Climate-related phenomena impact neurological and mental health through both direct and indirect mechanisms, including progressive temperature changes and more frequent extreme weather events. This has influenced the prevalence and geographic distribution of neurological disorders, affecting the public health landscape of these diseases. The primary mechanisms include thermal stress, neuroinflammation due to air pollution, ecological shifts that increase exposure to neurotropic infections, psychological stress, and disruptions to healthcare systems. These factors interact and amplify the risk of neurological diseases, including neurodegenerative, neuroinflammatory, cerebrovascular, neuroinfectious, and psychiatric conditions. The aim of this study was to synthesize evidence from peer-reviewed studies in major databases on the impact of climate change–related factors in the incidence, severity, and distribution of neurological and psychiatric disorders. Addressing the effect of climate change on these diseases requires improved healthcare strategies, scientific research, and climate change mitigation to protect brain health and reduce neurological disease burden.

## 1 Introduction

Climate change is an accelerating global crisis with profound implications for human health. The Earth’s mean surface temperature has risen by approximately 1.5°C since pre-industrial times, accompanied by more frequent extreme weather events (heatwaves, droughts, storms, wildfires, floods) (1). These environmental shifts are already impacting public health broadly, and emerging evidence indicates that neurological and psychiatric conditions are among those affected (2). Neurological disorders are a leading cause of disability and a major contributor to mortality worldwide, and severe mental illnesses, including schizophrenia, bipolar disorder, and severe major depression, carry a significantly reduced life expectancy (3). Understanding how climate change influences the incidence and severity of neurological and mental health conditions is therefore a critical scientific and public health challenge. Neurological and mental health conditions, while often distinct in etiology and clinical presentation, may also exist along a continuum in which biological, psychological, and social factors overlap. Certain disorders can present with both neurological and psychiatric manifestations, making them clinically indistinguishable at times. Recognizing this continuum provides the conceptual basis for addressing both domains together in this review. Recent studies have linked various climate-related factors, from extreme temperatures to altered ecosystems, with adverse neurological outcomes. However, the data are complex and sometimes sparse, complicated by heterogeneity in disease subtypes and regional differences (2). This review provides a comprehensive synthesis of peer-reviewed literature on the influence of climate change in the rise of neurological disease, encompassing neurodegenerative, neuroinflammatory, cerebrovascular, neuroinfectious, and psychiatric conditions. Key findings from epidemiologic studies and mechanistic research are discussed, highlighting both established patterns and areas of uncertainty. All information is drawn from credible medical and neuroscientific journals, with scientific sources cited throughout. For clarity, this review is organized into two main sections. The first examines climate change–related exposures and their general effects on neurological and mental health, including thermal extremes, extreme weather events, air pollution, vector-borne and infectious agents, and psychosocial stressors. The second presents disease-specific evidence, outlining how these exposures influence the incidence, progression, and outcomes of conditions such as dementia, stroke, multiple sclerosis, epilepsy, neuroinfectious diseases, headache disorders, and psychiatric disorders.

## 2 Methods

The literature search was conducted in March 2025 across PubMed, Embase, Scopus, Web of Science, and PsycINFO, combining climate-related terms (“climate change,” “global warming,” “extreme weather,” “heatwave,” “cold spell,” “wildfire smoke,” “air pollution,” “PM2.5,” “ozone,” “NO₂”) with neurological and psychiatric terms (“neurology,” “neurological disorders,” “neurodegenerative,” “stroke,” “multiple sclerosis,” “epilepsy,” “headache,” “psychiatric disorders,” “mental health,” “depression,” “anxiety,” “schizophrenia”) using Boolean operators and controlled vocabulary (MeSH/Entree). Inclusion criteria comprised peer-reviewed human studies (observational, interventional, time-series, or modeling analyses) and systematic reviews/meta-analyses reporting associations between climate-related exposures and neurological or mental health outcomes. Exclusion criteria included non–peer-reviewed materials, animal-only or *in vitro* studies (except when cited for mechanistic context), studies without neurological or mental health endpoints, conference abstracts without full text, and duplicates. Relevant references were also identified through citation tracking, and findings were narratively synthesized by exposure domain and disease-specific categories.

## 3 Environmental drivers and mechanistic pathways linking climate change to neurological and mental health

Climate change affects neurological health through multiple pathways, both direct and indirect. The major climate-related drivers impacting the nervous system include temperature extremes and heatwaves; extreme weather events; air pollution and poor air quality; vector-borne and infectious agents; and psychosocial stressors and lifestyle impacts. For orientation, Section 3 summarizes the exposure-specific epidemiologic and clinical signals, and Section 4 details the corresponding biological mechanisms.

### 3.1 Temperature extremes and extreme weather events

Unusually high ambient temperatures and heatwaves can precipitate acute neurological events and exacerbate chronic neurologic conditions. A meta-analysis revealed that heat wave days were associated with an increased risk of hospital admissions or visits (RR = 1.269; 95% CI: 1.030–1.564) and mortality due to mental disorders (RR = 1.266; 95% CI: 0.956–1.678), compared to non-heat wave days (4). Conversely, extreme cold spells have also been associated with cerebrovascular and psychiatric stress. In 2019, extreme low temperatures caused 474,000 stroke deaths globally, with higher rates in older males and affected individuals from central, and East Asia (5).

In addition, climate change is increasing the frequency of hurricanes, floods, wildfires, and droughts. Such events cause not only physical injury but also psychological trauma, and they interrupt medical care. Older adult patients with cardiovascular disease (CVD), who rely on pharmacies, are particularly vulnerable (6). Hong et al. found a 1.5% increase in stroke mortality (95% CI, 1.3–1.8%) for each interquartile range increase in particulate matter <10 μm in aerodynamic diameter (PM_₁₀_), and a 2.9% increase (95% CI, 0.3–5.5%) for ozone concentrations on the same day; these air pollutants are significant risk factors for acute stroke death (6, 7).

### 3.2 Air pollution and poor air quality

Warmer temperatures and environmental policies (e.g., increased fossil fuel combustion for cooling) can worsen air pollution (8). Outdoor air pollutants include particulate matter (PM), which is classified by size; with fine particles (PM_₂.₅_) being the most harmful. Other pollutants such as ozone (O_₃_), nitrogen dioxide (NO_₂_), volatile organic compounds (VOCs) and noxious gases have been strongly associated with stroke incidence, dementia, Parkinson’s disease, and headache frequency (9–11). Air pollution is considered a key mediator by which climate change impacts brain health, as inhaled pollutants trigger systemic inflammation and oxidative stress that can damage the nervous system (9, 10, 12). For instance, Peter et al. (11) found an association between PM_₂.₅_ and decline in cognitive performance and dementia. Mechanistic pathways underlying these associations are summarized in Section 4.2.

### 3.3 Vector-borne and infectious agents

Climate-driven shifts in ecosystems are expanding the range of vectors (mosquitos, ticks, etc.) and pathogens, leading to increased occurrence of neuroinfectious diseases. Warming temperatures and changing rainfall patterns have fuelled outbreaks of mosquito-borne viruses (e.g., West Nile, dengue, Zika) and the spread of tick-borne encephalitis (TBE) into new regions (11, 13). A time-series analysis shows increasing malaria prevalence at higher altitudes in Colombia and Ethiopia in recent years (14). These findings suggest that climate change is driving this upward shift, potentially raising malaria risk in previously unaffected highland regions of Africa and South America (14). A cohort study demonstrated that the incidence of TBE is linked to warm temperatures, which intensify virus replication (15). This highlights the importance of integrating weather-based forecasts to predict and manage vector-borne diseases.

### 3.4 Psychosocial stressors and lifestyle impacts

The chronic stress of living in a changing climate, including economic insecurity, displacement, and *eco-anxiety* about the future, is also an emerging risk to mental health (16, 17). High ambient temperatures have been linked not only to physiological stress but also to increased aggression and suicide rates. For every 1°C increase in monthly average temperature, suicide rates rise by 0.7% in U.S. countries and 2.1% in Mexican cities (16, 17). Moreover, climate change can indirectly affect neurological well-being by disrupting sleep patterns (e.g., hotter nights causing insomnia) and altering lifestyle behaviors (reduced outdoor activity, changes in diet), which in turn influence neurologic disease risk factors (18). For each 10 °C rising of ambient temperature, the odds of sleep insufficiency increased by 20.1%, while total sleep duration decreased by 9.67 min, with deep sleep declining the most, by 2.82% (19). These findings highlight climate change’s adverse impact on sleep health.

Furthermore, people with pre-existing neurological diseases are often especially vulnerable to these climate-related harms. For example, individuals with cognitive impairment or mobility limitations may be less able to adapt to extreme temperatures or evacuate during disasters; patients with dementia are exceptionally prone to heat-related illness or hypothermia during extreme weather, as their impaired judgment and awareness prevent appropriate behavioral adjustments, such as seeking cooler environments or adequate hydration (2, 4). Comorbid frailty and polypharmacy further compound this vulnerability. Likewise, neurologic medications and assistive devices can undermine normal thermoregulation, reducing tolerance to heat or cold (20, 21). In summary, climate change is creating new challenges for neurological health on a population level and is becoming an important public health matter. The following sections detail proposed mechanisms of action for this rise as well as disease-specific evidence of these impacts across a spectrum of neurological and psychiatric conditions.

## 4 Mechanisms of action for the rise of neurological disease

The associations described above between climate change and neurological/psychiatric outcomes are underpinned by a variety of biological and environmental mechanisms. Such mechanisms explain how climate-related factors translate into physiological stressors that can initiate or aggravate neurological/psychiatric pathology. Table 1 provides a summary of all these mechanisms.

**Table 1:** Mechanisms of action linking climate change to neurological/psychiatric disease.

Mechanism	Climate change factor	Neurological impact	Biological implication
Thermal stress and thermoregulatory failure	Climate extremes: hot and cold	Heat-induced neuroinflammation, cognitive dysfunction, exacerbation of neurodegenerative conditions (e.g., multiple sclerosis, Parkinson’s disease)	Altered ion channel function, changes in membrane excitability, increased seizure susceptibility, activation of microglia, impaired thermoregulation, and accelerated protein aggregation.
Air pollution and neuroinflammation	Increased particulate matter, ground-level ozone, and other climate-induced pollutants.	Cognitive decline, dementia, increased stroke risk, exacerbation of neurodegenerative diseases	Fine particulate matter (PM₂.₅) and other pollutants enter the bloodstream, cross the blood–brain barrier, and promote systemic oxidative stress and neuroinflammation.
Infectious disease ecology	Warmer temperatures, changes in precipitation patterns, and environmental shifts.	Neuroinfections (e.g., encephalitis, meningitis), autoimmune disorders (e.g., multiple sclerosis, Guillain-Barré syndrome)	Climate-driven changes in vector ecology (e.g., mosquitoes, ticks) enhance pathogen transmission, leading to increased exposure to neurotropic infections.
Psychological stress and neuroendocrine effects	Trauma, anxiety, chronic stress, and climate anxiety	Agitation, cognitive impairment, mood disorders, exacerbation of psychiatric illnesses (e.g., post-traumatic stress disorder, depression)	Activation of the hypothalamic–pituitary–adrenal (HPA) axis and prolonged cortisol elevation leading to hippocampal atrophy, disrupted neurochemistry, and altered emotional regulation.
Healthcare disruption and medication	Extreme weather events (e.g., heatwaves, storms)	Delayed or disrupted treatment, exacerbation of chronic neurological conditions (e.g., stroke, epilepsy, neuromuscular disorders)	Damage to healthcare infrastructure, medication degradation due to temperature fluctuations, and power outages preventing timely treatment delivery.

### 4.1 Thermal stress and thermoregulatory failure

The human brain and body function within a narrow temperature range, and both extreme heat and extreme cold challenge this homeostasis. High ambient temperatures can directly disrupt neural activity, heat has been shown to affect ion channel function in neurons, altering membrane excitability and synaptic transmission (22). For example, small increases in temperature can block conduction in demyelinated axons (explaining MS heat sensitivity) and can lower the seizure threshold by modifying neuronal firing patterns (23, 24). Heat also induces *systemic* responses; dehydration from sweating leads to hemoconcentration (increased blood viscosity and osmolarity) which raises the risk of thrombosis, contributing to ischemic strokes (25). Failure to maintain thermal homeostasis can impair neuronal function and disrupt cerebral blood flow and blood–brain barrier integrity, culminating, in severe cases, in cerebral oedema (22, 26). Heat strain triggers the release of inflammatory cytokines and heat-shock proteins that can breach the blood–brain barrier and activate microglia, potentially exacerbating neuroinflammation (9). In the context of neurodegeneration, chronic heat stress may accelerate protein misfolding or aggregation in the brain, although this is an area of active study. Cold exposure, on the other hand, activates sympathetic nervous system and hormonal responses (catecholamines, renin–angiotensin) that acutely raise blood pressure and heart rate (27, 28). This can precipitate hemorrhagic strokes or silent cerebrovascular damage. Cold can also slow nerve conduction and exacerbate spasticity or rigidity in conditions like Parkinson’s disease (PD) or spinal cord injury (29, 30). Notably, patients with neurological diseases often have impaired thermoregulatory defenses; for instance, spinal cord injury can impair shivering/sweating, and dementia or stroke can blunt the behavioral drive to seek shelter (29, 30). Thus, neurological patients are more likely to suffer the consequences of thermal extremes, creating a vicious cycle where climate events worsen their condition which in turn reduces their resilience to temperature stress. It should be noted, however, that not all heat exposure is uniformly harmful. Controlled thermal practices, such as sauna use, have been associated in some studies with possible cardiovascular and cognitive benefits, although the evidence is mixed and context dependent. These effects should not be conflated with the harmful and uncontrolled heat stress associated with climate change (31).

### 4.2 Air pollution and neuroinflammation

Air pollution constitutes a principal pathway through which climate change affects the nervous system; fossil fuel combustion generates particulate matter, nitrogen oxides, ozone, and other pollutants (32, 33). Warmer temperatures can intensify photochemical reactions that increase ground-level ozone, and climate change is linked to more wildfires that release fine particulates. These pollutants, when inhaled, have systemic effects; ultrafine particles (PM_₂.₅_ or smaller) can enter the bloodstream and even cross into the brain, directly depositing in neural tissue (32). Particulate pollution and ozone trigger systemic oxidative stress and inflammation (32). In the vasculature, this means endothelial dysfunction, a pro-coagulant state, and a propensity for atherosclerosis, all of which increase stroke risk (34). In the brain, particulate matter has been found in association with the presence of amyloid plaques and alpha-synuclein in autopsy studies, suggesting a role in neurodegenerative changes (32). Chronic exposure to polluted air is associated with cognitive decline and a higher incidence of dementia, likely via chronic neuroinflammation through activated microglial cells and cytokines that damage synapses and neurons over time (32, 33). Air pollutants can also irritate the respiratory tract and trigger reflexive autonomic responses (like surges in blood pressure or arrhythmias) that indirectly affect cerebral blood flow (35, 36). In headaches and migraines, inhaled pollutants may activate trigeminal nerve pathways or cause meningeal irritation, leading to headache pain (35, 36). Climate change driven increases in air pollution create a pro-inflammatory milieu that harms the central and peripheral nervous systems. Mitigating air pollution a co-benefit of climate action is therefore essential for protecting neurological health (12).

### 4.3 Alteration of infectious disease ecology

Mechanistically, higher temperatures can increase replication rates of viruses and shorten incubation periods in vectors (like mosquitoes and ticks), leading to higher viral loads transmitted to humans (13). Changes in rainfall and humidity create new breeding grounds (e.g., increased standing water after floods for mosquito breeding, or extended tick questing season due to milder winters) (13). Once pathogens infect humans, those that are neurotropic (such as arboviruses and certain bacteria/fungi) can directly invade the central nervous system; the immune response to these infections can cause acute neurological damage and sometimes initiate chronic autoimmune processes (e.g., some evidence links viral infections to triggering multiple sclerosis relapses or Guillain–Barré syndrome) (11, 37, 38). Climate-related migration and overcrowding can also facilitate the spread of infections like meningococcal meningitis in refugee camps or viral encephalitides in urban slums with poor sanitation (11, 37, 38). In essence, climate change serves as a catalyst for exposing human nervous systems to infectious insults that they might not have encountered previously, by removing geographical and seasonal barriers that once contained these pathogens.

### 4.4 Psychological stress and neuroendocrine effects

The stress imposed by climate change, whether acute trauma from disasters or chronic anxiety about the changing environment, translates into activation of stress pathways that affect the brain. Acute stress triggers the hypothalamic–pituitary–adrenal (HPA) axis to release glucocorticoids (cortisol) and catecholamines (39). While these are adaptive in the short term, chronic elevation (as seen in PTSD or prolonged anxiety) can be neurotoxic, leading to hippocampal atrophy and impairment in memory and mood regulation (39). Heat itself can act as a physiological stressor; experiments have shown that heat exposure elevates cortisol levels, which might contribute to agitation and confusion in vulnerable individuals (40). Moreover, high heat can cause sleep disturbance and poor sleep amplifies stress, creating a feedback loop. In psychiatric patients, stress from climate-related events can precipitate episodes of illness (for example, triggering a depressive episode or a psychotic break in schizophrenia) (41). On the other hand, climate anxiety represents a more nebulous but widespread mechanism; the awareness of climate threats can lead to chronic fear, especially in young people, which might manifest as generalized anxiety or depression (42). Neurobiologically, anxiety is linked to dysregulated amygdala activity and neurotransmitter imbalances resulting, in this case, in a constant worry about existential threats like climate change, keeping the brain in a hyper-vigilant, maladaptive state (43). This highlights that not all mechanisms are as tangible as heat or viruses, some are societal and psychological, yet they tangibly alter neurochemistry and behavior.

### 4.5 Disruption of healthcare and medication

Another mechanism, often overlooked, is the impact of climate change on healthcare delivery for neurological/psychiatric patients. Extreme weather can damage infrastructure (hospitals and roads), preventing patients from getting timely care (e.g., stroke thrombolysis or refilling epilepsy prescriptions) (44, 45). Heat can degrade certain medications or reduce their efficacy, as many drugs must be stored below 25°C; power outages from storms can shut down life-sustaining equipment for patients with advanced neuromuscular diseases (44, 45). These disruptions can turn stable chronic neurological conditions into acute life-threatening situations. Thus, climate resilience in healthcare systems is directly tied to patient neurologic outcomes; while this is not a biological mechanism within the body, it is a causal pathway from climate events to worsened neurological health at the population level (46).

It is important to note that these mechanisms often act in concert. For instance, a heatwave might simultaneously cause dehydration (raising stroke risk), worsen air pollution (adding inflammatory stress), disturb sleep (triggering seizures or cognitive issues), and induce anxiety, collectively amplifying health impacts. There are also feedback loops, for example, a person who suffers a stroke during a heatwave may then have reduced mobility and be even more vulnerable to the next heatwave. The interplay of multiple factors means the overall impact of climate change on the brain is not simply the sum of individual mechanisms, but a network of interacting stresses. This complexity underscores why research findings can sometimes appear inconsistent (e.g., different regions or subpopulations showing different dominant effects) (46, 47). Robust mechanistic studies, including experimental models and longitudinal human studies, are needed to further elucidate these pathways. Understanding mechanisms is vital for developing targeted interventions and policies to reduce these harmful factors to improve neurological health in a world where climate change will continue to be the norm (2).

## 5 Disease-specific evidence of the effects of climate change

### 5.1 Neurodegenerative diseases (dementia and Parkinson’s disease)

A growing body of evidence links climate change, particularly rising ambient temperatures, to worse outcomes in dementia. Epidemiological studies across various regions have shown that heatwaves and higher average temperatures correspond to increased hospitalizations and mortality among people with Alzheimer’s disease and other dementias (48, 49). In one study from New England, a 1.5°C increase in mean summer temperature was associated with a 12% rise in dementia-related hospital admissions (50). Similarly, during an extreme heat event in Madrid, admissions for Alzheimer’s disease surged by 23% when daily maximum temperature exceeded the local heatwave threshold by >1 °C (51). A study in the UK found that for each 1 °C increase above 17 °C, dementia admission rates rose by ~4.5% (52). These findings underscore the heightened vulnerability of dementia patients to heat stress. Cognitive impairment and memory loss in dementia can prevent patients from adequately protecting themselves (e.g., forgetting to drink water or adjust clothing), leading to dehydration or heat stroke (53). Moreover, some analyses suggest a U-shaped relationship, where both extreme heat and extreme cold increase dementia hospitalizations or deaths. Overall, unaccustomed temperature extremes appear to exacerbate dementia severity and precipitate acute crises in this population (49, 51). Aside from acute events, chronic climate-related exposures (such as long-term air pollution) have been linked to accelerated cognitive decline and higher dementia risk. Fine particulate pollution (PM_₂.₅_) exposure is associated with increased amyloid-*β* deposition and neuroinflammation in the brain, potentially contributing to neurodegeneration (53). An *in vitro* study found that PM_₂.₅_ exposure exacerbates amyloid-β-induced neuronal injury by elevating reactive oxygen species (ROS) levels and activating the NOD-like receptor pyrin domain-containing 3 (NLRP3) inflammasome in microglia, resulting in increased IL-1β production (54). Thus, climate change factors may act as both triggers of acute decompensation and drivers of long-term neurodegenerative processes in dementia.

Parkinson’s disease (PD) and related neurodegenerative disorders may also be influenced by changing environmental conditions, although data are more limited. An epidemiological study noted that the prevalence, mortality, or DALY (disability-adjusted life years) burden of PD tend to be higher in regions with warmer climates and higher recent warming indices, compared to cooler regions (54). PD shows greater sensitivity to climate warming than amyotrophic lateral sclerosis (ALS) /motor neuron diseases (MND) or Alzheimer’s, likely due to early hypothalamic degeneration, thermoregulatory dysfunction, and mitochondrial vulnerability in dopaminergic neurons unique to PD pathology (55). One major pathway by which climate change may affect PD is via air quality. Chronic exposure to air pollutants has emerged as a risk factor for PD; for example, long-term inhalation of traffic-related pollutants and fine particles is associated with a greater incidence of PD, higher PM_₁₀_ exposure significantly increases the risk of developing Parkinson’s disease (OR = 1.35) (33). Additionally, extreme heat can pose challenges for patients living with PD. Heat stress and dehydration may worsen blood pressure instability and fatigue in PD patients, who often have autonomic dysfunction (56). Climate change can worsen air pollution through increased wildfires and atmospheric stagnation, potentially increasing the neurotoxic burden on populations (57). There is also concern that climate-related disruptions (e.g., disasters) could interrupt access to PD medications or care, leading to symptom exacerbation. While direct causal links between climate variables and Parkinson’s disease progression remain to be clearly established, these indirect effects suggest that global warming and environmental change could aggravate the burden of PD. Further research is needed to disentangle how chronic neurodegenerative processes might be accelerated or triggered by the complex environmental changes underway.

### 5.2 Cerebrovascular diseases

**Figure 1:**
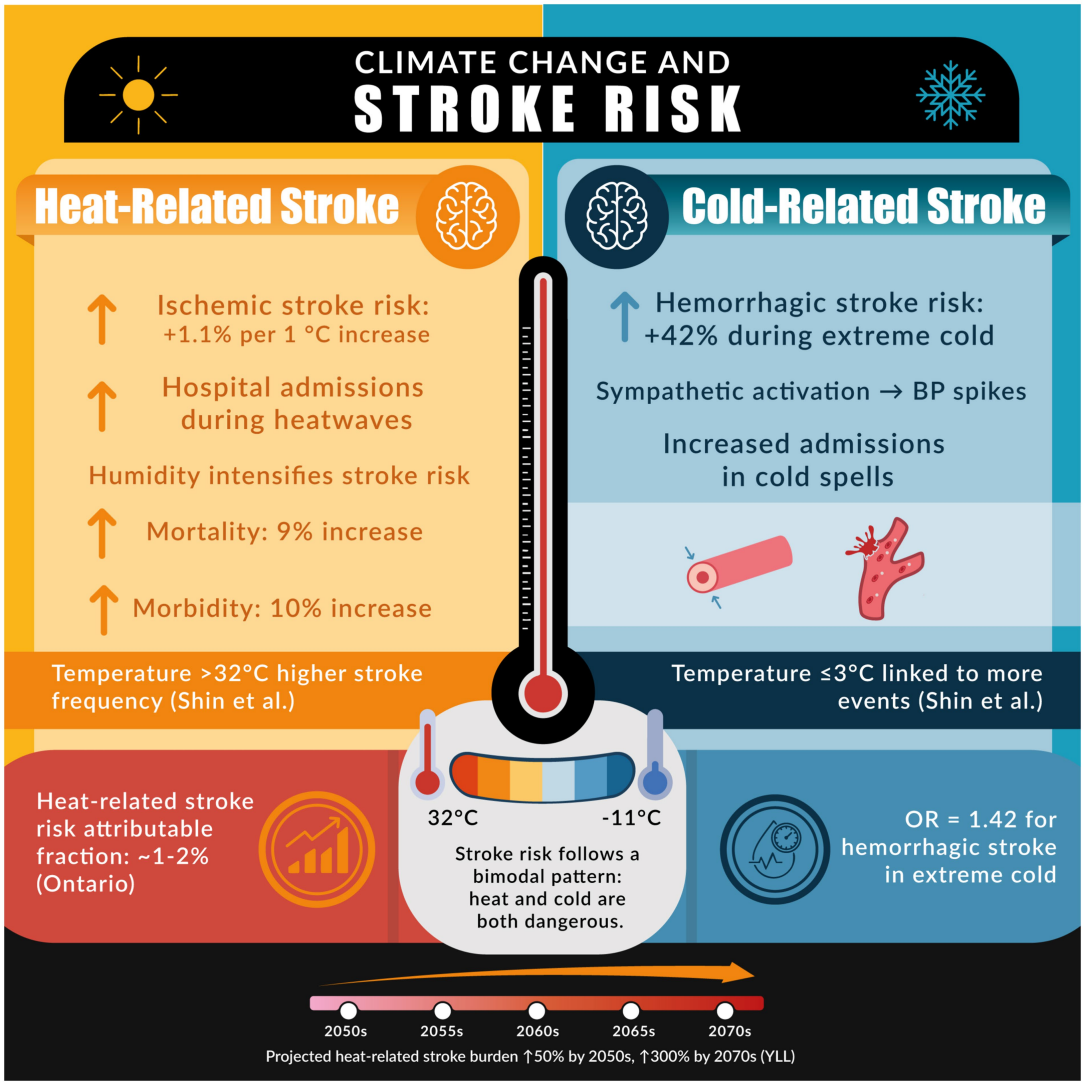
The figure illustrates the association between climate extremes and stroke risk, emphasizing how both elevated and reduced temperatures contribute to increased cerebrovascular events and related healthcare burdens.

Stroke is highly sensitive to environmental conditions, and numerous studies indicate that climate change is influencing stroke epidemiology. Both extreme heat and extreme cold have been associated with increased risk of stroke, though results have varied by region (57–64). Figure 1 provides an infographic showcasing the relationship between stroke (ischemic and hemorrhagic) and temperature. A comprehensive meta-analysis encompassing over 2 million stroke events found that short-term increases in ambient temperature were significantly associated with a rise in ischemic stroke incidence. A 1 °C temperature change was associated with a 1.1 and 1.2% significant increase in major adverse cerebrovascular events, for heat and cold, respectively (57). In many studies, hotter days and heatwaves correlate with higher rates of ischemic stroke and stroke-related hospital admissions (58). For example, Shin et al. found that acute stroke events were significantly more frequent when maximum daily temperatures exceeded 32 °C or dropped to ≤3 °C (*p* = 0.048), and when minimum temperatures fell below −11.0 °C (*p* = 0.020) (59). In a U.S.-based case-crossover study, the risk of ischemic stroke increased during periods of higher relative humidity, highlighting that humidity may intensify stroke risk (60). One analysis reported that days of extreme heat contributed an attributable fraction of ~1–2% of stroke admissions in a temperate region (Ontario, Canada) over a 17-year period (61). Large diurnal temperature swings, sudden weather changes where daytime and night-time temperatures differ widely, have also been linked to stroke occurrence; in one study from Shenzhen, China, an increased 24-h temperature range (exceeding ~5–8 °C) was estimated to contribute to 2–4% of first-time strokes (62). Additionally, high heat has implications for stroke outcomes: elevated ambient temperature during and after a stroke has been associated with worsened post-stroke morbidity and mortality. A pooled analysis found that high ambient temperatures were linked to a 10% increase in stroke morbidity (RR = 1.10, 95% CI: 1.02–1.18) and a 9% increase in stroke mortality (RR = 1.09, 95% CI: 1.02–1.17) (47).

Cold weather can be equally hazardous. Some investigations have found that stroke admissions rise following cold spells or during lower-than-average temperature periods (60, 63). Cold exposure may preferentially trigger hemorrhagic strokes by causing acute blood pressure elevations via sympathetic activation and vasoconstriction (26, 63, 64). A case-crossover study revealed that extreme low temperature was associated with 42% high risk of hemorrhagic stroke (OR = 1.42; 95% CI: 1.28–1.58), but not ischemic stroke (25, 64). The net impact of climate change on stroke is thus complex, potentially creating a *bimodal* risk pattern: unaccustomed heat and unaccustomed cold each pose dangers (25). Importantly, as global temperatures increase, many regions experience more frequent and intense heat extremes while still facing episodic cold snaps, thereby widening the range of temperature volatility. From a global health perspective, strokes are projected to rise in part due to climate trends. Modeling studies project that by mid-21st century, if climate change continues unabated, there will be an increase in years of life lost from stroke when factoring in rising temperatures along with demographic changes (64). However, under high emission scenarios, heat-related years of life lost (YLL) increase sharply, especially during summer over 150% in the 2050s and up to 300% in August by the 2070s (65).

Mechanistically, heat stress contributes to stroke through dehydration and hemoconcentration: profuse sweating and fluid loss thicken the blood and raise its viscosity, promoting thrombosis in cerebral arteries (6, 26). Heat also triggers systemic inflammatory responses and endothelial dysfunction, which can destabilize atherosclerotic plaques; in contrast, cold stress acutely raises blood pressure and may precipitate hemorrhagic stroke by inducing vascular rupture (25, 26). Aside from temperature, climate change–related air pollution plays a major role in stroke risk. Exposure to high levels of PM_₂.₅_ has been linked to both acute stroke events and chronic cerebrovascular disease. Indeed, an analysis from the Global Burden of Disease study attributed about 9% of stroke DALYs and 8–9% of stroke deaths worldwide to PM_₂.₅_ pollution (66, 67). Periods of extreme heat often coincide with spikes in air pollution (e.g., wildfire smoke or urban smog), compounding the risk. One study noted that during heatwaves accompanied by severe pollution and wildfires, the relative risk of death due to stroke was roughly threefold higher than normal (68). In summary, climate change is expected to aggravate stroke risk through a convergence of thermal stress and environmental pollution, posing a particular threat to the older adults and those with cardiovascular comorbidities who are less able to physiologically compensate for these stressors (69).

### 5.3 Neuroimmunological diseases

Multiple sclerosis (MS) is a chronic immune-mediated demyelinating disease that may be modulated by climate-related factors. While MS is classically more prevalent in higher latitudes (colder climates) due partly to lower ultraviolet exposure and vitamin D levels (70). Paradoxically, patients with MS often experience short-term worsening of symptoms in hot weather. Heat sensitivity in MS is well documented: up to 60% of individuals with MS report that elevated temperatures transiently exacerbate symptoms such as fatigue, weakness, and cognitive dysfunction; this phenomenon (Uhthoff’s phenomenon) occurs because increased core body temperature can further impair conduction in demyelinated nerve fibers (23). Consequently, heatwaves and unseasonal warm spells can reduce functional abilities in MS patients and potentially precipitate clinical relapses (70, 71). A study in Neurology found that warmer outdoor temperatures were associated with worse cognitive status in MS patients, corroborating patient-reported heat sensitivity (71, 72). Another analysis noted that high diurnal temperature variability was linked to increased emergency department visits for MS exacerbations, independent of absolute ambient temperature or air pollution. For instance, large swings in daily temperature were associated with a measurable uptick in MS-related hospital visits in one time-stratified case-crossover study (73).

Beyond temperature, other climate-linked factors can influence MS disease activity. Dehydration and heat stress may interfere with the effectiveness of MS therapies or increase circulating cytokines that drive inflammation. Conversely, some studies have hinted that extremely cold temperatures might also affect MS, for example, by increasing spasticity or triggering viral infections that lead to relapses; though heat effects are more consistently reported. Air pollution is another concern as exposure to high levels of particulate matter and nitrogen dioxide has been associated with increased MS relapse rates in some research (74, 75). Additionally, climate change might impact vitamin D synthesis (if people avoid sun exposure during heat extremes or if atmospheric changes alter UV levels), potentially affecting an important MS risk factor (76). While there is no evidence that climate change will alter the fundamental global distribution of MS in the short term, it is likely to add stressors that aggravate symptoms and possibly progression of disease. Maintaining adequate hydration, body cooling strategies, and air quality improvements are adaptation measures that could mitigate climate-related MS exacerbations. Given that MS typically affects young adults and can lead to long-term disability, understanding and managing these environmental triggers is crucial in a warming world.

### 5.4 Neuroinfectious diseases

**Figure 2:**
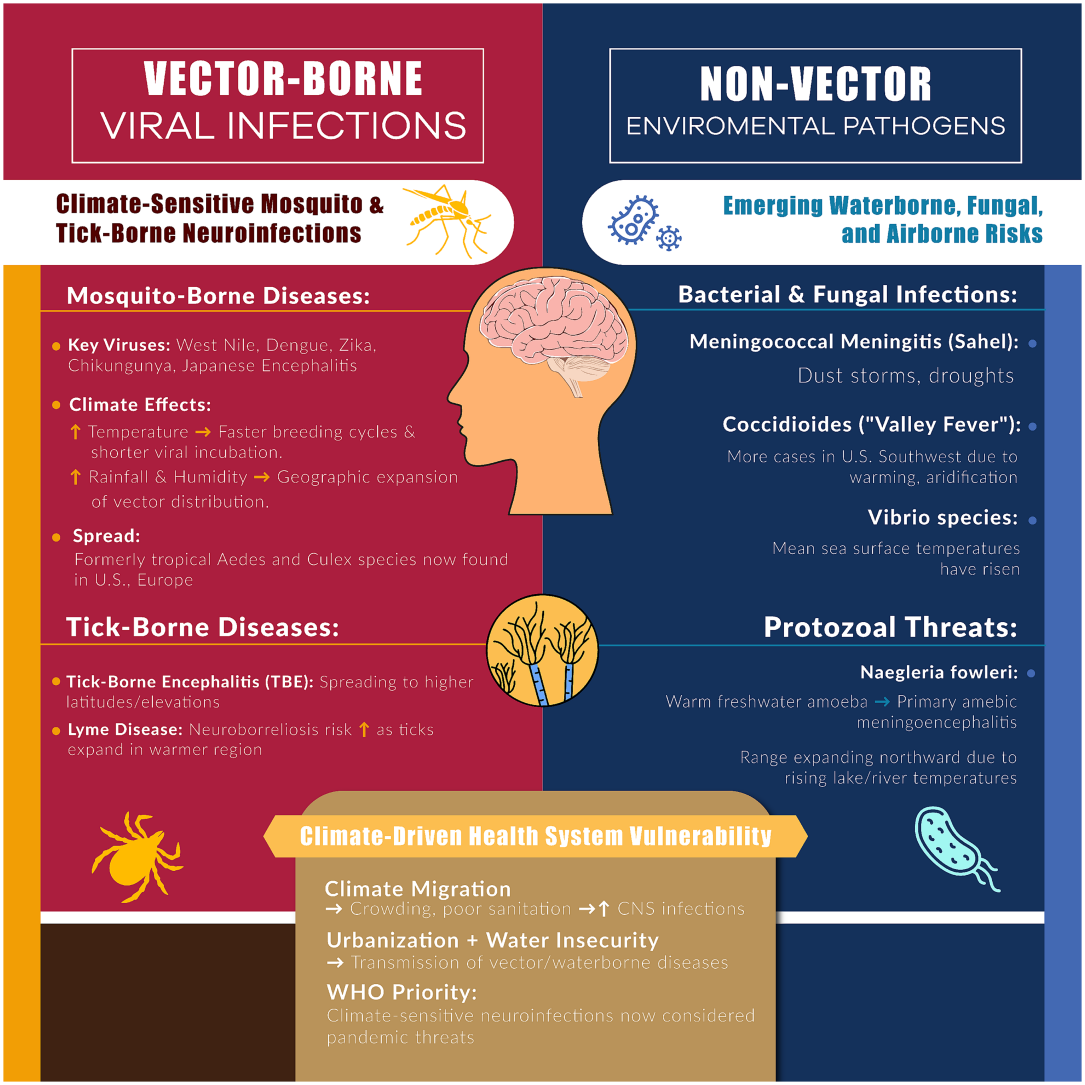
Climate change drives both vector-related (mosquito- and tick-borne) and non-vector related (water-, dust-, and soil-borne) infections, increasing neuroinfectious disease risks with climate change.

Climate change has a significant influence on the emergence and spread of infectious diseases that affect the nervous system. As temperatures rise and weather patterns shift, many pathogens and their vectors are expanding into new geographic areas, leading to increased incidence of neurotropic infections (76–88). Figure 2 provides and infographic summarizing the main factors related to neuroinfection and climate change. Vector-borne viral encephalitides are a prime example; mosquito-borne viruses such as *West Nile virus*, *Japanese encephalitis virus*, *dengue*, *chikungunya*, and *Zika* are all sensitive to climate conditions, including temperature, rainfall, and humidity (11, 77). Warmer climates shorten mosquito breeding cycles and viral incubation periods, which can amplify transmission (14, 76). West Nile virus, historically endemic to Africa and the Middle East, has now become established in North America and parts of Europe; its spread and the frequency of human neuroinvasive West Nile disease have been linked to milder winters, hotter summers, and drought conditions that favor the Culex mosquito vectors (78, 79). Similarly, dengue fever, the fastest-spreading tropical disease, has shown increased geographic range. Aedes mosquitoes that transmit dengue, Zika, yellow fever, and chikungunya are now found in parts of the United States and Europe, far beyond their former tropical range, due in part to climate warming and globalization (38, 80). These viruses can cause severe neurological complications; for instance, dengue can lead to encephalopathy, Zika virus causes congenital microcephaly and Guillain–Barré syndrome, and West Nile virus commonly causes encephalitis and meningitis in outbreaks (79, 81, 82). Certainly, climate models predict that as temperatures and humidity continue to change, the seasonal windows and geographic zones suitable for transmission of these arboviral encephalitis will expand further (83).

Tick-borne infections are also on the rise. Tick-borne encephalitis (TBE), a viral disease of the central nervous system (CNS), has extended its reach northward and to higher elevations in Europe and Asia as winters become milder and spring arrives earlier, prolonging the tick active season (83). Regions of higher latitude that previously saw little to no TBE are reporting new cases, consistent with climate-driven shifts (84). Lyme disease, caused by *Borrelia* spirochetes from ticks, is another infection whose incidence is influenced by warming trends and changes in ecosystems; while Lyme typically causes neurological issues in a subset of cases (neuroborreliosis), its overall burden provides trend setting information for vector-borne risk in temperate zones (85). Beyond arboviruses, bacterial and fungal infections show climate linkages too. In the African *meningitis belt*, outbreaks of meningococcal meningitis occur during the dry season when dust and low humidity damage nasal mucosa; changes in rainfall patterns and desertification could alter the timing and intensity of these epidemics (85). Some studies indicate that high dust conditions correlate with meningitis outbreaks in the Sahel, suggesting that climate change could influence meningitis incidence via increased dust storms or prolonged droughts (86). Waterborne pathogens that cause neurological disease are also a concern. The amoeba *Naegleria fowleri*, which causes rare but deadly primary amoebic meningoencephalitis, thrives in warm freshwater; as surface water temperatures rise, cases of this infection have appeared in previously cooler regions (87). Finally, *Vibrio* bacteria (some species can cause neuropathies via wound infections) and *Coccidioides* fungi (cause of Valley Fever meningitis) are likewise extending their range under warming and changing precipitation patterns (88, 89).

In summary, climate change impacts neuroinfectious diseases by altering pathogen lifecycles and expanding vector habitats. It also indirectly contributes via human behavioral and societal changes such as climate-related displacement, urbanization, and resource insecurity can lead to overcrowding and poor sanitation, which facilitate the spread of infections (including those causing CNS infections) (11, 90). Certainly, the World Health Organization has highlighted climate-sensitive infectious diseases (from viral hemorrhagic fevers to novel zoonoses) as priorities for monitoring, given their pandemic potential (91). For neurologists and healthcare systems, this means being prepared to diagnose and manage infections of the nervous system in regions and seasons where they were previously uncommon. Enhanced surveillance and vaccination (where available, e.g., TBE vaccine) will be key in mitigating these emerging threats.

### 5.5 Headache disorders

Migraine and other headache disorders appear to be susceptible to weather and climate influences, which may contribute to an uptick in headache burden as the climate changes. Many migraines sufferers report that environmental factors trigger or worsen their headaches; heat and temperature fluctuations are among the most reported triggers (91, 92). For example, an emergency department study found that a 5 °C increase in ambient temperature was associated with approximately a 7.5% higher risk of migraine-related hospital visits in the subsequent days (92). Similarly, days with large swings in temperature or abrupt barometric pressure changes have been linked to a higher likelihood of headache presentations. One study noted that decreases of 6–10 hPa from the standard atmospheric pressure (1,013 hPa) were most frequently associated with increased migraine and headache frequency, particularly in certain seasons (93). These findings suggest that the greater weather volatility expected with climate change (e.g., sudden hot-to-cold transitions or storms) could lead to more frequent headache exacerbations.

Air quality factors also play a role. Epidemiological research has identified associations between acute air pollution exposure and headache occurrences. A California-based study of nearly 90,000 cases found that higher average annual PM_₂.₅_ and NO_₂_ levels correlated with increased frequency of migraine emergency visits (94). Short-term spikes in pollutants like particulate matter (PM_₁₀_, PM_₂.₅_), ozone, and carbon monoxide have been linked with days of elevated headache clinic visits; ozone was found to be higher on days when patients presented with tension-type headaches (95). The biological mechanism is thought to involve pollution-induced inflammation and activation of trigeminal pain pathways. Climate change can worsen these exposures by prolonging smog events and increasing ground-level ozone formation during heatwaves; moreover, bright sunlight and high humidity, conditions influenced by climate, have been cited as migraine triggers in some individuals, possibly via perturbations in serotonin or other neurochemical systems (36, 96). While headaches are not typically life-threatening, they impose a substantial health burden and reduce quality of life. Migraine is among the leading causes of disability globally (97). Thus, even moderate climate-related increases in headache frequency can have large public health implications. Adaptation strategies might include public heat-health advisories that warn vulnerable individuals (like migraine patients) to stay hydrated and avoid excessive heat exposure. On an individual level, identifying weather triggers (using headache diaries) can help patients anticipate and possibly prevent some attacks. Further study is needed to refine our understanding of how chronic climate trends (as opposed to short-term weather) will affect headache disorders. Nevertheless, current evidence clearly indicates that a warming, more extreme climate is likely to aggravate migraines and other headaches, through both direct thermal effects and indirect air quality effects (92, 98).

### 5.6 Epilepsy and seizure disorders

Epilepsy, a condition characterized by recurrent seizures, may also be influenced by climate-related factors. Seizures can be triggered by systemic stressors and changes in the environment; researchers are beginning to uncover links between weather patterns and seizure frequency. Several studies have examined how meteorological variables affect epilepsy patients (24). Atmospheric pressure and humidity have emerged as potential factors; a case-crossover study of 604 adults with epilepsy in Germany found that a decrease of 10.7 hPa in atmospheric pressure was associated with a 14% increased risk of seizures (OR 1.14, 95% CI 1.01–1.28) and high relative air humidity (>80%) increased seizure risk by up to 48% with a 3-day lag (OR 1.48, 95% CI 1.11–1.96), whereas higher ambient temperatures were paradoxically associated with *reduced* seizure risk in that cohort (98). This suggests that certain individuals experience more seizures during stormy or humid weather, possibly due to hypoxia or changes in ion balance provoked by pressure shifts (99). Another study reported that “unstable weather” (sudden pressure changes) was associated with increased seizure frequency in about 40% of patients during spring, autumn, and winter, though interestingly only 7% noted this effect in the summer (100). These findings highlight that rapid weather fluctuations might precipitate seizures in a subset of susceptible people.

Heatwaves and temperature extremes have a more complex relationship with epilepsy. Some data indicate that extreme heat can provoke seizures or status epilepticus, potentially by causing dehydration, electrolyte disturbances, or sleep deprivation (101, 102). Heatstroke is known to cause acute brain insults that lower seizure threshold. Population studies show mixed results; heatwaves have been associated with a higher rate of hospital admissions for seizures/epilepsy in some reports (101), yet others have found that colder temperatures were linked to more emergency visits for seizures while hot days had little effect (102). A bimodal pattern has been suggested, where both very hot and very cold days can increase seizure risk for different patients (103). One explanation is that extreme heat often disrupts sleep, for example, nights that remain uncomfortably warm can cause insomnia. Sleep deprivation is a well-known seizure trigger, and thus prolonged heat could exacerbate epilepsy by fragmenting patients’ sleep cycles. Indeed, rising night-time temperatures under climate change are thought to be particularly detrimental, as they rob the body of the nocturnal cooling needed for restorative sleep (104, 105).

Other indirect pathways include the effect of climate on anti-epileptic drug levels and medical infrastructure. High ambient temperatures or disasters might compromise medication storage (many drugs, including anti-seizure medications, have reduced efficacy if stored above certain temperatures) (105). Interruptions in healthcare delivery during climate disasters (floods, hurricanes) could lead to medication lapses, triggering breakthrough seizures. Additionally, prolonged droughts or heat may increase incidence of renal stones or infections, which in turn can provoke seizures in susceptible patients (24). While these aspects are less studied, they highlight how climate events can intersect with epilepsy management.

In summary, climate change is expected to present new challenges for epilepsy control. More volatile weather with frequent swings may lead to more triggers for those whose seizures are barometric-sensitive. Increasing heat, especially if accompanied by poor sleep or dehydration, could aggravate seizure disorders for some patients (102). However, individual responses vary, and further research (preferably prospective studies) is needed to better predict which patients are at risk and how to protect them. Physicians may consider advising patients about maintaining hydration, avoiding sleep deprivation during heatwaves, and having contingency plans for medication supply during extreme weather events (101). Importantly, what is evident is that epilepsy does not exist in isolation from the environment; climate stressors can and do influence neurological excitability and seizure thresholds.

### 5.7 Psychiatric and neurodevelopmental disorders

Psychiatric conditions, including mood disorders, anxiety disorders, schizophrenia, and others, are profoundly affected by the environment, and climate change is increasingly recognized as a mental health threat. Unlike many of the neurological diseases discussed above, the impact of climate on psychiatric disorders often manifests through acute stress and slowly accumulating psychosocial pressures (16, 106). Nevertheless, physiologic effects of heat and climate extremes play a role as well. In this review, we use the term severe mental illness (SMI) to refer specifically to schizophrenia, bipolar disorder, and severe major depression. These conditions are associated with high disability, premature mortality, and increased healthcare utilization, and patients with SMI are disproportionately vulnerable to climate-related stressors, particularly extreme heat, due to both medication-related thermoregulatory impairment and adverse social determinants (106, 107).

A consistent finding across global studies is that higher temperatures are associated with an increase in acute mental health crises. Time-series data from Bern, Switzerland (1973–2017) encompassing nearly 90,000 psychiatric hospitalizations showed that for every 10°C rise in daily mean temperature, psychiatric hospital admission rates increased by about 4%—and this effect was even more pronounced in patients with schizophrenia and neurodevelopmental disorders (108). In the United States, an analysis of ~3.5 million emergency department visits (2010–2019) found 8% higher odds of a mental health emergency on days of extreme heat (defined relative to local temperature distributions) (41). These findings align with historical observations that psychiatric disturbances can have seasonal patterns. Heat may exacerbate underlying psychiatric symptoms (for example, causing agitation or delirium in vulnerable individuals) (106). Moreover, certain psychiatric medications (like antipsychotics and some antidepressants) impair thermoregulation or cause dehydration, making patients with serious mental illness less heat-tolerant; tragically, during heatwaves, patients with schizophrenia or bipolar disorder are known to have higher rates of heat-related death than the general population (20, 107). On the other end of the spectrum, very cold temperatures and shorter daylight can worsen mood disorders (as seen in seasonal affective disorder), though the balance of evidence suggests heat is the more immediate concern with climate change (109). Importantly, extreme heat has been linked to increased suicide rates. A study across multiple countries found that a 1°C increase in average monthly temperature was associated with roughly a 0.7% rise in suicide (19). Projections indicate that unabated climate change could increase suicide rates by 1–2% in some regions by mid-century, compounding the existing suicide public health burden (17). The proposed mechanisms include heat-induced neurochemical changes that affect mood and impulse control, as well as the general stress caused by discomfort.

Climate change-driven disasters (hurricanes, floods, wildfires, and droughts) can acutely and chronically impact mental health (109–114). Immediately following disasters, rates of acute stress disorder, post-traumatic stress disorder (PTSD), anxiety, and depression tend to spike in affected communities (110). For instance, communities hit by catastrophic floods or fires report high levels of anxiety and PTSD that may persist for years (110). Chronic disaster exposure can also erode social supports and economic stability, leading to long-term psychological distress. There is evidence that even prenatal exposure to extreme stress (for example, a fetus in utero during a disaster experienced by the mother) can influence neurodevelopment and increase the risk of developmental or psychiatric issues later in life (111). Wildfires pose a double threat, as they cause trauma and displacement while also polluting the air with fine particles that have been linked to cognitive and mood changes (112). Droughts and heatwaves can lead to economic hardship (especially in agrarian communities) and have been associated with rises in depression and suicide among affected farmers (113). In sum, climate change acts as a *threat multiplier* for mental health, often hitting disadvantaged populations the hardest.

Beyond direct disaster trauma, the psychological burden of climate change includes the phenomenon of climate anxiety, defined as a chronic fear and worry about environmental damage and future risks; this is particularly observed in children and young adults who are increasingly aware of climate change (42). Large-scale surveys have found that most of the youth worldwide report being at least moderately worried about climate change and feel sad, anxious, or helpless about its impacts (114). While climate anxiety is not a clinical disorder per se, intense levels of worry can exacerbate or precipitate conditions like generalized anxiety disorder or depression. Therapists are reporting more patients struggling with *eco-grief* or despair related to environmental loss; this mental health toll, though harder to quantify, represents an indirect but pervasive impact of climate change on well-being (115). Overall, psychiatric disorders are clearly sensitive to the changing climate with high temperatures correlating with more psychiatric emergencies (41). There are also complex interactions; for example, heat might worsen aggression or psychosis, leading to increased violence or hospitalizations as one Swiss study noted that the effect was especially significant in neurodevelopmental conditions and schizophrenia (108). Furthermore, social factors (housing quality, community resilience, and healthcare access) can buffer or worsen these outcomes. Notably, a UK study of patients with severe mental illness, dementia, or substance misuse found a 4.9% increase in the risk of death for each 1 °C rise above the 93rd percentile of local temperature, underscoring that the most vulnerable patients face lethal risks on the hottest days (107).

In psychiatry as well as in neurology, climate change’s impacts are multifaceted. It is not only the dramatic events (like environmental disasters) but also the subtler shifts (warmer nights, longer allergy seasons, etc.) that can cumulatively strain mental health. This calls for integrating mental health support into climate adaptation and disaster response planning (115, 116). It also highlights the need for interdisciplinary research bridging climatology, neuroscience, and psychology to fully unravel how environmental changes translate into brain and behavior changes.

## 6 Discussion

The evidence gathered in our review shows that climate change is not a distant or abstract threat to neurological health, its effects are already measurable across a broad spectrum of diseases. From the acute impacts of heatwaves on stroke and mental health crises, and psychiatric morbidity to the insidious spread of neurotropic infections, climate-related factors are influencing when, where, and how neurological diseases manifest. The data show that certain trends are relatively clear; extreme heat is broadly deleterious, linked with increased incidence of stroke, higher hospitalization rates for dementia and mental illness, more headaches, and disrupted sleep leading to seizure exacerbations (46). At the same time, other climate stressors add layers of risk; for example, wildfires and pollution spikes often accompany heat, jointly worsening cerebrovascular and neurodegenerative conditions (32). Changes in vector ecology are introducing infections to immunologically naive populations, as seen with West Nile virus in temperate zones and the northward march of tick-borne encephalitis (15, 79). These multi-faceted impacts underscore that climate change is creating a new landscape of neurological risk factors.

Despite these advances in understanding, there are significant challenges and gaps that need to be tackled. One major challenge is the heterogeneity of findings across different studies and regions. Some studies report heat-related increases in stroke, while a few report cold-related effects; some data suggest minimal impact on certain conditions while others show large effects (117). This heterogeneity can be attributed to differences in study design, population adaptation, and local infrastructure. Populations vary in their baseline climate (a “hot” day in Finland is very different from a “hot” day in India), and thus the relative impact of a given temperature anomaly will differ (118, 119). Moreover, many studies are retrospective and not originally designed to probe climate effects, leading to confounding factors and inconsistencies. There is also a geographical research bias, meaning much of the climate-neurology data comes from high-income countries, whereas low- and middle-income regions (which often experience more severe climate impacts and have the largest neurological disease burdens) are underrepresented (120). This limits our ability to generalize findings globally. Another consideration is that neurological diseases are diverse and multifactorial. Each disorder (be it Alzheimer’s, stroke, MS, or schizophrenia) has its own set of primary drivers (genetics, lifestyle, infections, etc.), and climate is an added layer of influence. Distinguishing a climate signal from background variability requires large datasets and careful analysis. For instance, stroke rates are influenced by hypertension prevalence and healthcare access; demonstrating a climate effect means adjusting for these factors, something not all studies manage uniformly (25, 121). Additionally, many neurological conditions have lagging outcomes, exposures in early life might influence disease risk decades later (e.g., developmental impacts or cumulative exposure effects); few studies have the longitudinal design to capture such long-term influences of climate change.

Nevertheless, a converging theme is that climate extremes, events outside the range of usual, tend to precipitate neurological crises (64). Populations have some capacity to adapt to their normal climate (e.g., people in tropical regions are physiologically acclimatized to heat to an extent), but climate change is pushing beyond historical norms. Unprecedented heatwaves, hundred-year floods happening frequently, and unfamiliar disease exposures mean communities are often unprepared. Neurologic patients, especially those who are older adults or disabled, are disproportionately affected by these unanticipated extremes (122, 123). The concept of resilience becomes important, just as we talk about making infrastructure resilient to climate change, we must bolster the resilience of patients and healthcare systems. This can include early warning systems (for heat-related illness, poor air quality, etc.), community cooling centers during heatwaves, ensuring backup power for medical equipment, and integrating mental health support in disaster response (124). There are also co-benefits to be harnessed. Actions taken to combat climate change can directly benefit neurological health. For example, reducing fossil fuel combustion will lower air pollution, which could translate to fewer strokes, improved cognitive function, and lower risk of Parkinson’s and dementia down the line (46). Urban planning that encourages green spaces may mitigate heat island effects and promote physical activity and social engagement, which are protective against cognitive decline and depression. Conversely, poorly planned climate mitigation strategies could have unintended consequences, so a thoughtful approach is required (for instance, some regions might rely more on biomass burning for energy if coal is reduced, but that could worsen local air quality unless clean energy is provided) (125, 126).

Another vital aspect is awareness and education. Neurologists and psychiatrists are beginning to recognize climate change as a factor in patient health. Initiatives like the “Hot Brain” conference (the first dedicated meeting on climate change and neuroscience in 2024) highlights a growing interdisciplinary effort to address these issues (127). Training healthcare providers to consider environmental factors in their clinical assessment (e.g., asking about heat exposure in a patient with new neurologic symptoms, or recognizing PTSD after climate disasters) will improve diagnosis and management. At the policy level, health agencies should include neurological outcomes in climate impact assessments and adaptation plans (128). Traditionally, discussions of climate and health focus only on infectious diseases and heat stroke; this review underscores that brain health deserves a seat at the table in climate policy deliberations and that more conditions should be considered. Beyond individual-level risk factors, structural and social determinants play a critical role in shaping neurological and psychiatric vulnerability to climate change. Displacement, resource scarcity, and limited healthcare access disproportionately burden marginalized communities, amplifying inequities in outcomes. Furthermore, phenomena such as climate anxiety and ecological grief, particularly evident among youth and populations directly exposed to environmental loss underscore that climate change acts not only as a biological stressor but also as a social multiplier of risk (114, 115). Integrating these determinants is essential for advancing planetary health and health equity.

In summation, it is evident that climate change represents a *new determinant of neurological disease*. It acts synergistically with traditional risk factors to increase disease burdens and health disparities (since poorer communities often lack the resources to adapt, they suffer more). The rise of neurological diseases in a warming world is not an inevitable outcome; timely mitigation of climate change and proactive adaptation can substantially reduce the impact. This includes reducing greenhouse gas emissions in line with global targets to limit warming, and concurrently investing in public health measures to protect vulnerable populations from the climate impacts already locked in. The neurological community has a role to play in advocating for these changes, conducting relevant research, and preparing healthcare systems for what lies ahead. As the data show, the stakes are high, the health of our brains and minds may well depend on the actions we take to address climate change now.

### 6.1 Future directions

Given the evidence, it is imperative for clinicians, researchers, and policymakers to act on several fronts. First, neurological health should be incorporated into climate adaptation strategies; this means safeguarding patients (e.g., ensuring continuity of care during disasters, establishing heat action plans for those with cognitive impairment, and improving air quality) and educating communities about the neurological risks of climate extremes. Second, more research is needed to fill knowledge gaps, particularly in understanding long-term impacts and in regions that are under-studied, so that we can better predict and prevent climate-related neurological harm. Third, aggressive climate change mitigation is essential. Reducing greenhouse gas emissions will not only slow global warming but also confer direct benefits like cleaner air and fewer extreme events, thereby protecting against some of the neurological risks outlined in our review (129).

### 6.2 Limitations

The limitations of our review, and the studies included therein, are that current research include a paucity of prospective studies, limited mechanistic experiments in human models, and uncertainty in projecting future disease burdens. Mental health evidence is additionally constrained by heterogeneous outcome definitions, under-ascertainment after extreme events, and short follow-up. Very few studies project how, for example, Alzheimer’s disease incidence might change by 2050 under climate scenarios, yet such projections would be valuable for planning healthcare resources (130). Interdisciplinary research bridging climate science and neurology, and psychiatry is urgently needed to fill these gaps. Encouragingly, climate scientists and neurologists have started collaborating on models (for instance, using climate data to predict future stroke mortality) (65); extending these approaches to mental health outcomes should improve attribution and preparedness. As more data accumulate, confidence in attributing changes in neurological and mental-health patterns to climate change will grow.

## 7 Conclusion

Climate change exerts a growing and multifaceted influence on neurological health. Our review underscores that the nervous system, far from being exempt, is increasingly vulnerable to a cascade of climate-related stressors; from acute cerebrovascular events and neurodegeneration to psychiatric and broader mental health morbidity, diverse conditions show susceptibility to environmental perturbations, including extreme temperatures, air pollution, shifting infectious patterns, and psychosocial trauma. We have synthesized evidence indicating that climate change alters not only disease incidence and distribution but also clinical course and severity. Climate change affects brain health across the neuropsychiatric spectrum. Evidence synthesized here indicates substantial mental health impacts—acute stress responses around extreme events and exacerbations of mood, anxiety, and psychotic disorders under adverse environmental conditions. Accordingly, mental health should be explicitly integrated into surveillance, health-system preparedness, and adaptation strategies to address the full burden on brain and behavioral health. Mechanistically, pathways involving thermoregulatory stress, neuroinflammation, endocrine disruption, and pollutant-mediated toxicity intersect with existing vulnerabilities in at-risk populations. The cumulative effect is likely synergistic rather than merely additive. In short, brain health is inseparable from environmental health; as neurological disease burden rises together with ecological instability, strategic adaptation becomes highly important. Recognizing this interplay is relevant for the design of resilient healthcare systems, effective public health strategies, and informed climate policy. Addressing climate-linked neurological/psychiatric risk should not be speculative but rather an active, evidence-supported issue requiring interdisciplinary action.
